# The *PknI* and *DacB2* double deletion mutant of *Mycobacterium tuberculosis* leads to alteration of cell morphology and susceptibility to antibiotics

**DOI:** 10.1186/1471-2334-14-S3-E22

**Published:** 2014-05-27

**Authors:** Srinivasan Kandasamy, Sujatha Narayanan

**Affiliations:** 1 Department of Immunology, National Institute for Research in Tuberculosis (NIRT) (formerly TRC), ICMR, Chetpet, Chennai-600031, India

## Background

*Mycobacterium tuberculosis* is a slow growing infectious pathogen. It takes twenty hours for a single cell to divide into two. Its cell division is complex involving a number of proteins. Although, the physiological roles of several serine/threonine phosphorylation connected to cell division and peptidoglycan synthesis have been studied the exact mechanism is not clear. *PknI* and *DacB2* located in a same cluster have been shown to play a role in cell division and cell wall synthesis. The aim of this present study was to construct the double deletion mutant (DKO) of *PknI* and *DacB2* and study the effect on cell morphology and antibiotic susceptibility.

## Methods

Specialized phage transduction method was used to construct DKO strain of *PknI* and *DacB2*. The cell morphology was observed in solid agar plate, light microscopy and electron microscopy. The MIC was determined by resazurin based microplate assay.

## Results

The DKO was confirmed by PCR and southern blotting methods. The light and electron microscopy study revealed that DKO showed irregular shape and smoother colonies in comparison to *M. tuberculosis* H37Rv. The DKO was more susceptible to isoniazid compared to *M. tuberculosis* H37Rv and DKO showed the same sensitivity pattern as H37Rv strain to other drugs.

## Conclusion

In the present study, we have successfully constructed a novel DKO strain of *Mycobacterium tuberculosis*. *PknI* and *DacB2* were found to have a role in maintaining cell morphology and isoniazid resistance.

